# In frame exon skipping in *UBE3B* is associated with developmental disorders and increased mortality in cattle

**DOI:** 10.1186/1471-2164-15-890

**Published:** 2014-10-12

**Authors:** Heli Venhoranta, Hubert Pausch, Krzysztof Flisikowski, Christine Wurmser, Juhani Taponen, Helena Rautala, Alexander Kind, Angelika Schnieke, Ruedi Fries, Hannes Lohi, Magnus Andersson

**Affiliations:** Department of Production Animal Medicine, Faculty of Veterinary Medicine, University of Helsinki, Paroninkuja 20, 04920 Saarentaus, Finland; Lehrstuhl für Tierzucht, Technische Universitaet Muenchen, Liesel-Beckmann-Straße 1, 85354 Freising, Germany; Lehrstuhl für Biotechnologie der Nutztiere, Technische Universitaet Muenchen, Liesel-Beckmann-Straße 1, 85354 Freising, Germany; Department of Veterinary Biosciences, Research Programs Unit, Molecular Neurology, University of Helsinki and Folkhälsan Research Institute, Haartmaninkatu 8, 00290 Helsinki, Finland

**Keywords:** Kaufman oculocerebrofacial syndrome, Increased juvenile mortality, Intellectual disability, Structural malformation, *UBE3B*, Bovine, Splicing cite mutation

## Abstract

**Background:**

Inherited developmental diseases can cause severe animal welfare and economic problems in dairy cattle. The use of a small number of bulls for artificial insemination (AI) carries a risk that recessive defects rapidly enrich in the population. In recent years, an increasing number of Finnish Ayrshire calves have been identified with signs of *p*tosis, *i*ntellectual disability, *r*etarded growth and *m*ortality, which constitute an inherited disorder classified as PIRM syndrome.

**Results:**

We established a cohort of nine PIRM-affected calves and 38 unaffected half-siblings and performed a genome-wide association study (GWAS) to map the disease to a 700-kb region on bovine chromosome 17 (p = 1.55 × 10^-9^). Whole genome re-sequencing of an unaffected carrier, its affected progeny and 43 other unaffected animals from another breed identified a G > A substitution mutation at the last nucleotide of exon 23 in the ubiquitin protein ligase E3B encoding gene (*UBE3B*). *UBE3B* transcript analysis revealed in-frame exon skipping in the affected animals resulting in an altered protein lacking 40 amino acids, of which 20 are located in the conserved HECT-domain, the catalytic site of the UBE3B protein. Mutation screening in 129 Ayrshire AI bulls currently used in Finland indicated a high carrier frequency (17.1%). We also found that PIRM syndrome might be connected to the recently identified AH1 haplotype, which has a frequency of 26.1% in the United States Ayrshire population.

**Conclusion:**

We describe PIRM syndrome in cattle, which is associated with the mutated *UBE3B* gene*.* The bovine phenotype resembles human Kaufman oculocerebrofacial syndrome, which is also caused by mutations in *UBE3B*. PIRM syndrome might be connected with the recently identified AH1 haplotype, which is associated with reduced fertility in the US Ayrshire population. This study enables the development of a genetic test to efficiently reduce the high frequency of mutant *UBE3B* in Ayrshires, significantly improving animal health and reducing economic loss.

**Electronic supplementary material:**

The online version of this article (doi:10.1186/1471-2164-15-890) contains supplementary material, which is available to authorized users.

## Background

Inherited developmental diseases can cause severe animal welfare and economic problems in dairy cattle breeding. Where a limited number of sires are used for artificial insemination (AI), recessive genetic defects can rapidly enrich in cattle populations. Examples include degenerative axonopathy in Tyrolean Grey cattle [1] and compromised growth and regulation of the inflammatory response in Belgian Blue cattle [2]. We recently reported foetal growth retardation and stillbirth in half the progeny of an Ayrshire bull carrying a genetic deletion in the *MIMT1* gene [3]. Identification of the causative mutation and development of a gene test enable such carrier animals to be excluded from breeding.

In the past few years, an increasing number of Finnish Ayrshire calves have been identified with a combination of severe symptoms including ptosis, intellectual disability, retarded growth and mortality, a disorder classified as PIRM syndrome. Bovine PIRM resembles the human autosomal recessive neurodevelopmental disorder Kaufman oculocerebrofacial syndrome, also known as blepharophimosis-ptosis-intellectual disability syndrome (MIM 615057, MIM 244450), caused by ubiquitin protein ligase E3B *(UBE3B)* mutations [4–6].

*UBE3B* belongs to the family of ubiquitin E3 ligases involved in protein ubiquitination, a post-translational protein regulation pathway that plays a key role in several biological processes during organogenesis and neurodevelopment. Mutations of other E3 ligases are associated with a variety of human developmental diseases. Increased copy number of the *HUWE1* gene (HECT, UBA and WWE domain containing 1, E3 ubiquitin protein ligase) causes cognitive impairment in males (MIM 300706) [7]. Missense mutations in *CBL* (Cbl proto-oncogene, E3 ubiquitin protein ligase) cause impaired growth, developmental delay, cryptorchidism and predisposition to juvenile myelomonocytic leukemia [8, 9]. The best known example of these defects is Angelman syndrome characterised by intellectual disability, absence of speech, motor dysfunction and seizures (MIM 105830) caused by loss of function of the imprinted gene *UBE3A* (ubiquitin protein ligase E3A) [10, 11].

Here we report that PIRM syndrome (*p*tosis, *i*ntellectual disability, *r*etarded growth and *m*ortality) in cattle is associated with an exon skipping mutation in *UBE3B* and this mutation is present at high frequency in the sample of AI bulls tested. Moreover, our data suggest an association between the recently identified AH1 haplotype [12] and PIRM. Our findings have practical implications for cattle breeding and provide a new model for human Kaufman oculocerebrofacial syndrome.

## Results

### PIRM syndrome in the Ayrshire population

**Figure 1:**
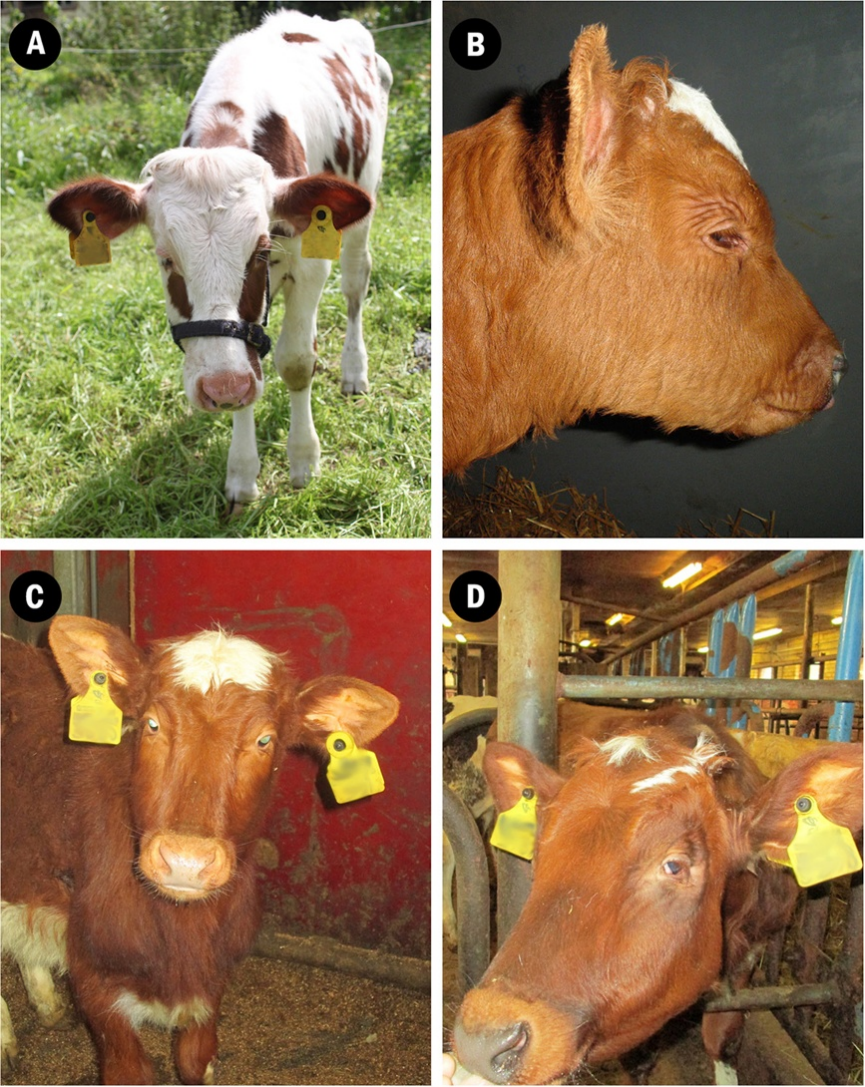
**Phenotypic manifestation of PIRM syndrome.** Most obvious facial feature in PIRM-syndrome is the ptosis. Abnormal large upper eye lid gives a characteristic appearance to affected animals, almost like they were constantly sleepy **(A-D)**. Affected animals also suffered also from hypotonia **(A and B)** or were smaller than other age matched calves **(C and D)**. In addition owners reported problems with learning and dependency of special care. All animals were euthanized soon after the pictures were taken.

Farmers and breeding counsellors have reported an increasing number of calves with developmental defects including ptosis, post-natal growth retardation and increased juvenile mortality in the Finnish Ayrshire population between 2011 and 2014. Some affected calves also suffered from feeding problems, minor structural changes of the head and muscular hypotonia (Figure 1, Additional file 1: Table S1). Many affected calves failed to thrive and died at a very young age if not euthanized before. Breeders also reported learning difficulties indicating intellectual disability. For example calves had difficulties learning how to use feeding buckets. Surviving calves required special care during the neonatal period and later showed growth retardation. Usually, farmers culled affected animals before breeding. Both sexes were equally affected. The phenotype has been defined as PIRM syndrome according to its typical features (*p*tosis, *i*ntellectual disability, *r*etarded growth, *m*ortality). The analysis of pedigree records of affected animals and their close relatives indicated an autosomal recessive mode of inheritance.

### PIRM syndrome maps to a 713 kb segment on bovine chromosome 17

**Figure 2:**
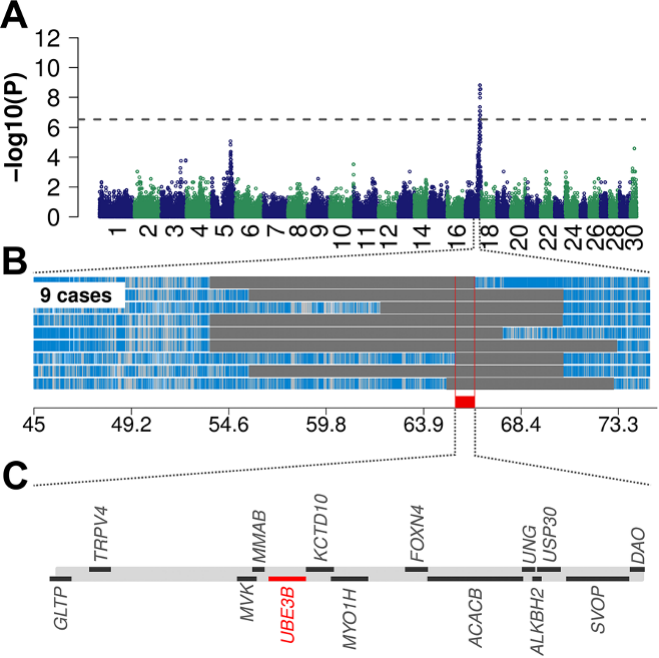
**The PIRM syndrome maps to chromosome 17 in the Ayrshire cattle population.** Association of the affection status in nine affected and 37 unaffected Ayrshire animals **(A)**. P-values were obtained by calculating Fisher exact tests of allelic association. Autozygosity mapping in nine affected animals **(B)**. Blue and pale blue represent homozygous genotypes (AA and BB), heterozygous genotypes (AB) are displayed in light grey. The solid grey bars represent segments of extended homozygosity in nine affected animals. The red bar indicates the common segment of homozygosity. The shared segment of homozygosity encompasses 14 transcripts among them *UBE3B* **(C)**. The full list of genes within the 713 kb segment is presented in Additional file 2: Table S2.

To identify the genomic region associated with PIRM syndrome, nine affected and 37 unaffected calves descended from one AI bull were genotyped with a bovine high-density genotyping array. After quality control, genotypes for 623,881 SNPs were phased using *Beagle's* hidden Markov model based algorithm. The haplotypes obtained were then used in a genome wide association study. A sliding window-based approach was used to compare haplotype frequency in cases and controls, which revealed a strong association on bovine chromosome 17 (Figure 2A). The most significant association (P = 1.55 × 10^-9^) resulted from four adjacent haplotype windows located between 65,659,074 bp and 65,981,740 bp. To narrow down the associated region, the genotypes of affected animals were screened for segments of homozygosity. A common 713 kb region (65,645,831 bp - 66,358,629 bp) with extended homozygosity was present in all affected animals while none of the unaffected animals showed homozygosity, suggesting a recessive pattern of inheritance. The risk haplotype encompasses 14 genes (Figure 2B-C, Additional file 2: Table S2).

### A synonymous mutation in UBE3B coincides exactly with PIRM syndrome

To identify the underlying mutation responsible for PIRM, the whole genomes of an obligate carrier and one of its affected progeny were sequenced to average read depths of 10.4 and 10.1. Sequence depth analysis across the 713 kb haplotype did not reveal large structural elements in the PIRM associated region (Additional file 3: Figure S1). To help identify candidate causal mutations, we used re-sequencing data from 43 previously sequenced Fleckvieh animals [13]. There is no evidence that the PIRM syndrome occurs in breeds other than Ayrshire. Thus, the mutation causing the PIRM syndrome should not segregate among the sequenced Fleckvieh animals. Multi-sample variant calling in the 713 kb segment of extended homozygosity yielded genotypes for 1825 polymorphic sites (1684 SNPs, 141 Indels, Additional file 4: File S1). Among these only four SNPs were compatible with recessive inheritance that is homozygous for the reference allele in 43 FV animals, heterozygous in the carrier bull and homozygous for the non-reference allele in the affected animal. The functional effects of those variants were predicted based on annotation of the UMD3.1 bovine genome assembly [14]. One of the four compatible variants was intergenic, two were located in intronic regions of *TRPV4* and *UBE3B*, and one variant was located in the coding region of *UBE3B* (rs475678587, Chr17:65,921,497 bp) (Table 1). However, two of the four variants (rs440561578, rs467377722) segregated also among 191 non-Fleckvieh animals that were sequenced in the context of the 1000 bull genomes project [15] and can be therefore excluded as being causative. In conclusion, only a coding and an intronic variant of the *UBE3B* gene segregate with the PIRM syndrome.

**Table 1:** **Variants compatible with recessive inheritance**

Chromo-some	Chromosomal position (bp)	NCBI assay ID	Reference allele	Alternative allele	Affected gene	Effect
17	65,696,110	rs440561578	C	T	*TRPV4*	intronic
17	65,850,261	rs467377722	C	T	---	---
17	65,905,778	rs463975690	A	G	*UBE3B*	intronic
17	65,921,497	rs475678587	G	A	*UBE3B*	splicing site

Four SNPs compatible with recessive inheritance were located in the 713 kb segment of extended homozygosity. The functional annotation of the identified polymorphisms was obtained based on the UMD3.1 gene prediction [21].

We annotated the bovine *UBE3B* gene and found that it consists of 32 exons, of which exons 1 to 6 are non-coding (Additional file 5: Table S3). Variant rs475678587 is a G > A polymorphism in the third base of codon 692 that would appear to cause a synonymous substitution, p.E692E in exon 23 of *UBE3B*. The rs475678587 polymorphism was validated by Sanger sequencing in the carrier bull, its nine affected and 37 unaffected descendants. In addition, nine new cases and two control animals were also genotyped by a KASP genotyping assay. All 18 affected animals were homozygous for the rs475678587 A variant, whereas the unaffected animals were either heterozygous or wild type (reference allele). The suspected carrier bull and 23 of its descendants were heterozygous.

### Incidence of the rs475678587 A variant in AI bulls

To determine the frequency of the rs475678587 A variant in the Finnish Ayrshire population we analysed 129 AI bulls and found a 17.1% carrier frequency. Assuming random mating, this would generate one affected calf per 138 offspring. Of those tested, 29 AI bulls had a known haplotype status for AH1. The AH1 haplotype was perfectly associated with the rs475678587 A mutation in this cohort: All 11 bulls that were carriers of the rs475678587 A mutation carry also the AH1 and 18 wild type bulls did not have the AH1.

### The rs475678587 A mutation affects splicing of UBE3B

**Figure 3:**
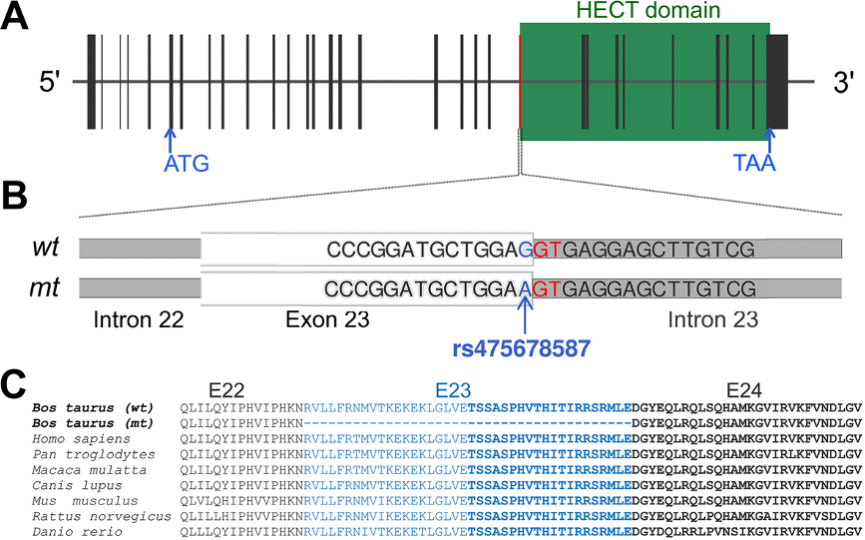
**The rs475678587 A mutation in** ***UBE3B*** **affects splicing.** Genomic structure of bovine *UBE3B* **(A)**. Bovine *UBE3B* consists of 32 exons (vertical bars) and its translation starts in exon 7. The position of the HECT-domain was determined according to [18]. Wildtype (wt) and mutant (mt) sequence of exon 23 **(B)**. To improve readability, only part of the genomic sequence is shown. The rs475678587 A mutation affects the very last nucleotide of exon 23 (B) within the highly conserved HECT-domain. Multi-species alignment of the UBE3B protein sequence **(C)**. Alternating colour indicates different exons (22–24). Bold type indicates the initiation of the HECT domain.

**Figure 4:**
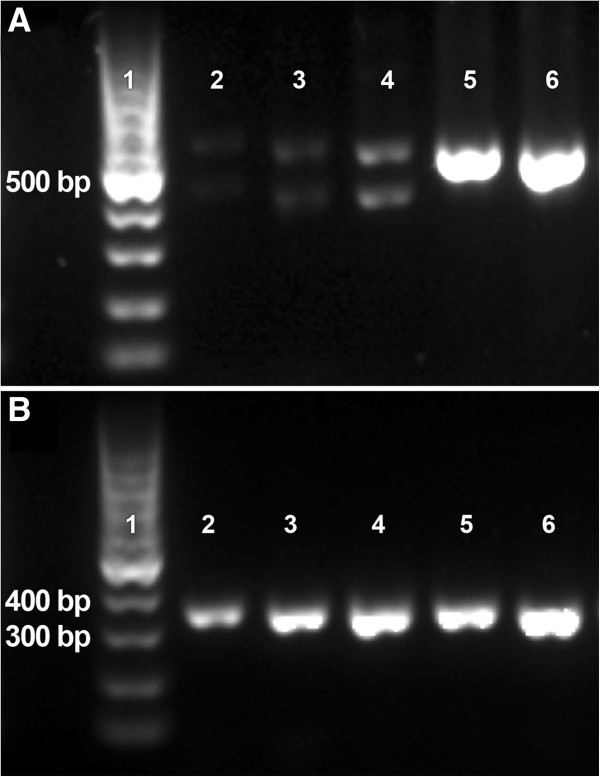
**Mutation rs475678587 causes partial skipping of exon 23 in** ***UBE3B*** **.** Agarose gel electrophoresis of the RT-PCR products reveals that in addition to normal band affected animals also express smaller fragment with primers that flanked the exon 23 **(A)**. Amplicons obtained with primers that flanked the exon 27–29 show no difference between affected and unaffected animals **(B)**. 1 is molecular weight marker. Cerebellum samples 2–4 are from PIRM affected animals and 5 and 6 are from unaffected animals.

The rs475678587 G > A polymorphism is located at the junction of exon 23 and intron 23 (Additional file 5: Table S3, Figure 3B) and could therefore affect RNA splicing. The effect of the mutation on *UBE3B* splicing was investigated by RT-PCR in samples of cerebral cortex, tectum, hippocampus, cerebellum, lung, liver, heart, kidney, spleen, and ovary from three affected and two unaffected animals, using two primer pairs. Primer pair 2 (Additional file 6: Table S4), which flanked exon 23, resulted in the amplification of two fragments from the affected animals and only one fragment from the unaffected animals (Figure 4A). Sequencing of the smaller RT-PCR product revealed in-frame exon 23 skipping. The other fragment of expected size corresponded to the reference sequence (the University of Maryland reference sequence UMD3.1, [16]) with the exception of the rs475678587 A variant that was detected in affected animals.

The *UBE3B* gene was expressed in all examined tissues and the altered splicing pattern was observed in all tissues of affected animals, excluding tissue specificity. RT-PCR data obtained with primer pair 2 suggested lower relative expression levels of *UBE3B* in affected animals, but RT-PCR results obtained with primer pair 3 (Additional file 6: Table S4) that flanked exons 27–29 showed no difference between unaffected and affected animals (Figure 4B).

### In silico analysis predicts in-frame deletion of 40 amino acids

Amino acid alignment of the normal and mutated proteins showed that loss of exon 23 results in an in-frame deletion of 40 amino acids between residues 652–692, deleting 20 amino acids from the E2 binding subdomain of HECT in *UBE3B* (Figure 3). Three-dimensional modelling of the mutated HECT domain revealed a lack of one α-helix structure and considerable structural differences compared to the normal HECT domain (Additional file 7: Figure S2).

## Discussion

We report a new congenital developmental syndrome in the Ayrshire breed and indicate its association with an exon skipping mutation in the *UBE3B* gene. The complex disease phenotype is characterized by ptosis, intellectual disability, retarded growth and mortality, and named PIRM accordingly. Genetic analyses based on across-breed comparisons efficiently shortlisted the number of candidate causative variants and ultimately identified an in-frame exon skipping mutation in *UBE3B*. Similar genetic comparisons approaches recently enabled the rapid identification of disease-causing alleles in cattle [15, 17]. Exon skipping results in a partial truncation of the HECT-domain in the UBE3B protein, likely compromising its function.

Loss-of-function mutations in *UBE3B* cause severe neurodevelopmental disorders in humans such as developmental delay, intellectual disability and characteristic facial dysmorphisms, e.g., ptosis, blepharophimosis and telecanthus. Affected individuals also suffer from severe growth retardation, hypotonia, microcephaly, neonatal respiratory distress, difficulties with feeding and gastrointestinal tract and hypocholesterolemia (MIM 615057, MIM 244450, [4–6]). Mice engineered to lack *UBE3B* expression (UBE3B^-/-^) display severe growth retardation and significant reduction of total cholesterol and lathosterol. Additionally, increased embryonal and perinatal lethality was reported for UBE3B^-/-^ mice [5]. Strikingly similar pathological signs were observed in PIRM Ayrshire cattle. *UBE3B* mutations in heterozygous form do not cause any clinically detectable symptoms in cattle, which accords with studies in human and mouse.

The functional role of *UBE3B* in different biochemical pathways is still unknown. Our RT-PCR results show that, as in human [18, 19] and mouse [5, 20], bovine *UBE3B* is expressed in several tissues. Earlier results show that UBE3B is involved in protein degradation under stress conditions. *UBE3B* mRNA expression was found to be up-regulated in damaged areas of chicken inner ear after noise trauma [19] and the *UBE3B* orthologue *OXI-1* in *C. elegans* is associated with oxidative stress-response [5, 21]. Increased sensitivity toward oxidative stress might be associated with neuronal dysfunction [5]. In addition *UBE3B* is connected with total cholesterol in plasma [22] and importantly *UBE3B* lesions in human and mouse are associated with hypocholesterolemia [4, 5]. Cholesterol is essential for normal development. Decreased cholesterol synthesis might be particularly harmful to the brain, which cannot utilize circulating cholesterol because of the blood–brain barrier [5, 23].

Both wild type and mutated transcripts are expressed in PIRM-affected animals. This is most likely due to ineffective splicing. Similar in-frame exon skipping mutations have been found in other developmental disorders, such as anophthalmia/microphthalmia [14], cholesteryl ester storage disease [15] and dystrophic epidermolysis bullosa [16]. Bioinformatic analysis predicted that the mutated UBE3B protein lacks 40 amino acids, including 20 in the N-terminal E2 subdomain of the highly conserved HECT-domain. HECT domains consist of two subdomains, the large N-terminal subdomain which contains the E2 binding site, and the small C-terminal subdomain that harbours the catalytic Cys residue required for ubiquitin transfer to the substrate. The E2 binding site mediates the interaction with the cognate E2 ubiquitin-conjugating enzyme.

Cooper et al. [12] recently reported a segment with homozygous haplotype deficiency on bovine chromosome 17 (AH1) in Ayrshire cattle, which encompasses the rs475678587 polymorphism. In our study, the rs475678587 A mutation was perfectly associated with AH1. Our findings suggest that in-frame exon skipping in *UBE3B* might be connected to the lack of AH1 homozygous animals. Missing homozygosity for AH1 could be associated with juvenile mortality, growth retardation and developmental disorders of PIRM-affected calves. Reduced fertility in AH1 carrier matings [12] additionally could imply embryonic losses that agree with findings of UBE3B deficiency in mice [5]. The estimated frequency of the AH1 haplotype was 26% in the US Ayrshire herd [12] even higher than the cohort we examined, and thus a matter of considerable concern to cattle breeders and ranchers. Our study does however provide an avenue for further investigation.

## Conclusion

We showed here that bovine PIRM syndrome, which resembles human Kaufman oculocerebrofacial syndrome, is associated with a *UBE3B* mutation. The ubiquitin/proteosome system (UPS) regulates many cellular signalling pathways such as the Notch and Hedgehog that play a key role in neurogenesis [19]. Therefore, deregulation of UPS can affect neuronal function and lead to neurological disorders [20]. Our data support the importance of UBE3B protein for normal development in mammals. Moreover, PIRM syndrome is the first inherited disease of the ubiquitin-dependent pathway identified in cattle. The bovine PIRM model could provide the basis for comparative studies with mouse and human.

The rs475678587 A mutation was found in 17.1% of analysed Ayrshire AI bulls and is probably associated with the prevalent AH1 haplotype and therefore can cause serious economic problems for breeders and animal welfare problems if AI carrier bulls are used for breeding.

## Methods

### Ethics statement

Blood sampling and clinical studies were carried out according to standard Finnish veterinary protocols. Tissue samples were collected after slaughter or euthanasia. All animal experiments were approved by the Animal Ethics Committee of the State Provincial Office of Southern Finland (ESAVI/641/04.10.07/2014).

### Sampling

Samples of venous blood were collected (with EDTA) from 18 affected and 41 unaffected animals for mutation mapping and frequency analysis. Most of the clinical examinations and symptom observations were done in farms by local veterinarians, farmers and breeding counsellors. Semen samples of 129 randomly selected bulls that are currently or have recently been used for AI in Finland were collected to gain an estimate of the carrier frequency in AI bulls. Tissue samples were collected from the cerebral cortex, tectum, hippocampus, cerebellum, lung, liver, heart, kidney, spleen, and ovary of three PIRM affected and two control animals for RNA expression analysis.

### High- density genotyping, quality control and haplotype inference

Genomic DNA of forty-seven half sibs (9 affected, 38 unaffected) were isolated using a semi-automated Chemagen extraction robot (Chemagen Biopolymer-Technologie AG) and genotyped with the Illumina BovineHD Bead chip comprising 777,962 SNPs. Genotypes were called using Illumina BeadStudio data analysis software and default parameters. Quality control was carried out using the PLINK v1.07 whole genome association analysis toolset [24]. Chromosomal positions of SNPs were determined on the basis of the UMD3.1 bovine genome assembly [16]. 1224 Y-chromosomal, 343 mitochondrial and 1735 SNPs with unknown chromosomal position were excluded from further analysis. The genotypes of one unaffected animal were omitted because genotyping failed in more than 10% of SNPs. We further excluded 7235 SNPs because genotyping failed in more than 10% of individuals, and 149,129 SNPs with minor allele frequency less than 0.5%. The final dataset contained 46 animals (9 affected, 37 unaffected) and 623,881 SNPs with an average per individual call-rate of 99.38%. *Beagle* genetic analysis software (version 3.2.1) [25] was used with its default settings for imputation of sporadically missing genotypes and for haplotype inference.

### Haplotype-based association study

A sliding window of 80 adjacent SNPs (corresponding to an average haplotype length of ~0.33 Mb) was shifted along the entire genome in steps of 15 SNPs. Within each window, haplotypes with frequency >5% were tested for association with PIRM syndrome using Fisher's exact test of allelic association.

### Generation of sequence data

Genomic DNA was prepared from semen of the supposed carrier bull and blood from one of his affected progeny by standard protocols using proteinase K digestion and phenol-chloroform extraction. Paired-end libraries were prepared using the paired-end TruSeq DNA sample preparation kit (Illumina inc., San Diego, CA, USA) and sequenced using the HiSeq 2000 system (Illumina, San Diego, CA, USA). The read length was 101 bp. Resulting reads were processed during the sequencing step with the Illumina BaseCaller. Reads were aligned to the University of Maryland reference sequence (UMD3.1) [16] using the Burrows Wheeler Aligner (version 0.6.1-r104) [26] with default parameters. Individual files in SAM (Sequence Alignment/Map) format were converted into BAM (Binary Alignment/Map) format using *SAMtools* (version 0.1.18) [27]. Duplicate reads were identified and marked with the MarkDuplicates command of *Picard* [28]. Net fold coverage was 10.4 for the bull and 10.1 for its affected descendant.

### Variant calling and imputation

Polymorphic sites in the region of interest (Chr17:60,000,000 bp – 70,000,000 bp) including short insertions and deletions were called for the two animals sequenced together with 43 previously sequenced Fleckvieh (FV) key ancestors [13] using the multi-sample approach implemented in *mpileup* of *SAMtools* along with *BCFtools* [27]. Read duplicates (see above) and positions with coverage of more than 720 reads (corresponding to 2 × N samples x average coverage) were not considered in variant calling. *Beagle* (version 3.2.1) phasing and imputation (see above) was used to improve the primary genotype calling by *SAMtools*.

### Identification of a candidate causal variant

Multi-sample variant calling yielded genotypes for 54,240 variants (49,245 SNPs and 4995 Indels) within the 10 Mb region of bovine chromosome 17. Among those, 1825 variants were located in the 713 kb segment (65,645,831 bp - 66,358,629 bp) of extended homozygosity. To identify mutations compatible with the presumed recessive mode of inheritance, these sites were filtered for variants that met three conditions: (i) the affected animal was homozygous for the reference allele, (ii) the presumed heterozygous carrier bull was heterozygous and (iii) all 43 sequenced FV animals were homozygous for the reference allele, as there are no indications that a similar phenotype is segregating in the FV population. Functional effects of the candidate causal variants were predicted based on gene annotation of the UMD3.1 bovine genome assembly [14].

### Manual re-annotation of bovine UBE3B gene

The genomic structure of *UBE3B* was predicted based on the University of Maryland UMD3.1 bovine genome sequence assembly [16] and the Dana-Farber Cancer Institute bovine gene index release 12.0 [29] using *GENOMETHREADER* software tool [30]. The *GENOMETHREADER* output was viewed and edited using the Apollo sequence annotation editor [31].

### Validation of rs475678587 polymorphism

PCR primer pair 1 was designed with Primer 3 [32] for exon 23 of bovine *UBE3B* to scrutinise the rs475678587 polymorphism by classical Sanger sequencing in the carrier bull and its nine affected and 37 unaffected descendants. Genomic PCR products were sequenced using a 3730 × l DNA Analyser (Applied Biosystems) and data analysed with the Variant Reporter v1.0 program (Applied Biosystems). All primer pairs are listed in Additional file 6: Table S4.

### Analysis of p.E692E-polymorphism in a larger cohort

DNA from semen samples of 129 AI bulls was extracted with a Chemagen extraction robot (Chemagen Biopolymer-Technologie AG). In brief, 200 μl diluted and frozen semen was washed twice with 1000 μl PBS (centrifugation for 5 min at 10,000 g) and resuspended in 500 μl lysis buffer (Chemagic DNA Blood Kit special, article No. CMG-703-1) containing 2 μl proteinase K (20 mg/ml) and 20 μl DTT (1 M). Samples were incubated overnight at 55°C and extraction continued according to the manufacturer’s instructions with 1 ml isolation buffer and 150 μl elution volume. DNA from blood samples of nine new affected calves and two control animals for the expression analysis was extracted as described earlier. Polymorphism frequency analysis was studied using KASP (Kompetitive Allele Specific PCR) reagents (LGC) and a 7500 Fast Real-Time PCR instrument (Applied Biosystems) according to the manufacturer's instructions. For quality control, every run included two samples of each rs475678587 polymorphism group determined earlier by Sanger-sequencing. From the tested 129 AI bulls 29 had genotype tested AH1 record provided individually in the Canadian Dairy Network website [33].

### Expression analysis

Total RNA of tissue samples was isolated with RNeasy Mini Kit (Qiagen) and converted to cDNA with High Capacity RNA-to-cDNA Kit (Applied Biosystems) according to the manufacturer's instructions. *UBE3B* gene expression was studied by RT-PCR analysis with two primer pairs designed with Primer 3 [32]. PCR products of cerebral cortex and liver obtained with primer pair 2 were extracted from gel with GenElute™ Gel Extraction Kit (Sigma-Aldrich) and the DNA sequence determined. The identity of PCR products obtained with primer pair 3 was confirmed by sequence analysis from cerebral cortex and liver samples.

### Bioinformatic analysis

The effect of the p.E692E-polymorphism on mRNA splicing was predicted using the web based tool ESEfinder 3.0 [34, 35]. UBE3B protein alignment was carried out using the ClusterW2 tool [36] and the effect of the absence of exon 23 on protein structure was investigated using the protein homology recognition engine V2.0 - PHYRE2 [37].

### Availability of supporting data

The sequencing data of 43 Fleckvieh animals are publically available in the Sequence Read Archive of NCBI (http://www.ncbi.nlm.nih.gov/sra) under accession numbers SRX527690-SRX527732. Four compatible variants are submitted to the The Single Nucleotide Polymorphism database of NCBI (http://www.ncbi.nlm.nih.gov/snp) under accession numbers rs440561578, rs467377722, rs463975690 and rs475678587.

## Electronic supplementary material

Additional file 1: Table S1: Major clinical features of 18 affected animals. (DOCX 21 KB)

Additional file 2: Table S2: Gene content within the segment of extended homozygosity on bovine chromosome 17. The gene content was assessed based on the UMD3.1-assembly of the bovine genome sequence. A total of 14 transcripts/genes were identified within the segment of extended homozygosity. (DOCX 21 KB)

Additional file 3: Figure S1: Average read depth in the PIRM-associated region. Each dot represents the average read depth of 5 adjacent variants displayed as deviation from the average sequence coverage for the affected animal (A), the supposed carrier of the mutation (B) and an unaffected Fleckvieh animal (C). The grey shaded area represents the segment of extended homozygosity including *UBE3B.* (TIFF 2 MB)

Additional file 4: File S1: Genotypes for 1825 variants located in the disease-associated region in 43 Fleckvieh (FV1-FV43) and 2 Ayrshire (AYR_case, AYR_heterozygous) animals. Details of the variant calling pipeline are described in the material & methods section. (ZIP 478 KB)

Additional file 5: Table S3: Prediction of the genomic structure of the bovine *UBE3B* gene. (DOCX 20 KB)

Additional file 6: Table S4: PCR primers. (DOCX 19 KB)

Additional file 7: Figure S2: Three dimensional modelling of the UBE3B HECT domain. The photos show a PHYRE2 analysis of the normal UBE3B protein (A) and lacking 40 amino-acid of which 20 belong to E2 subdomain of HECT (B). E2 subdomain is shown in red. (TIFF 907 KB)

## References

[1] Drogemuller C, Reichart U, Seuberlich T, Oevermann A, Baumgartner M, Kuhni Boghenbor K, Stoffel MH, Syring C, Meylan M, Muller S, Muller M, Gredler B, Solkner J, Leeb T (2011). An unusual splice defect in the mitofusin 2 gene (MFN2) is associated with degenerative axonopathy in Tyrolean Grey cattle. PLoS One.

[2] Sartelet A, Druet T, Michaux C, Fasquelle C, Geron S, Tamma N, Zhang Z, Coppieters W, Georges M, Charlier C (2012). A splice site variant in the bovine RNF11 gene compromises growth and regulation of the inflammatory response. PLoS Genet.

[3] Flisikowski K, Venhoranta H, Nowacka-Woszuk J, McKay SD, Flyckt A, Taponen J, Schnabel R, Schwarzenbacher H, Szczerbal I, Lohi H, Fries R, Taylor JF, Switonski M, Andersson M (2010). A novel mutation in the maternally imprinted PEG3 domain results in a loss of MIMT1 expression and causes abortions and stillbirths in cattle (Bos taurus). PLoS One.

[4] Flex E (2013). Loss of function of the E3 ubiquitin-protein ligase UBE3B causes Kaufman oculocerebrofacial syndrome. J Med Genet.

[5] Basel-Vanagaite L, Dallapiccola B, Ramirez-Solis R, Segref A, Thiele H, Edwards A, Arends MJ, Miro X, White JK, Desir J, Abramowicz M, Dentici ML, Lepri F, Hofmann K, Har-Zahav A, Ryder E, Karp NA, Estabel J, Gerdin AK, Podrini C, Ingham NJ, Altmuller J, Nurnberg G, Frommolt P, Abdelhak S, Pasmanik-Chor M, Konen O, Kelley RI, Shohat M, Nurnberg P (2012). Deficiency for the ubiquitin ligase UBE3B in a blepharophimosis-ptosis-intellectual-disability syndrome. Am J Hum Genet.

[6] Basel-Vanagaite L, Yilmaz R, Tang S, Reuter MS, Rahner N, Grange DK, Mortenson M, Koty P, Feenstra H, Farwell Gonzalez KD, Sticht H, Boddaert N, Desir J, Anyane-Yeboa K, Zweier C, Reis A, Kubisch C, Jewett T, Zeng W, Borck G (2014). Expanding the clinical and mutational spectrum of Kaufman oculocerebrofacial syndrome with biallelic UBE3B mutations. Hum Genet.

[7] Froyen G, Belet S, Martinez F, Santos-Reboucas CB, Declercq M, Verbeeck J, Donckers L, Berland S, Mayo S, Rosello M, Pimentel MM, Fintelman-Rodrigues N, Hovland R, Rodrigues dos Santos S, Raymond FL, Bose T, Corbett MA, Sheffield L, van Ravenswaaij-Arts CM, Dijkhuizen T, Coutton C, Satre V, Siu V, Marynen P (2012). Copy-number gains of HUWE1 due to replication- and recombination-based rearrangements. Am J Hum Genet.

[8] Niemeyer CM, Kang MW, Shin DH, Furlan I, Erlacher M, Bunin NJ, Bunda S, Finklestein JZ, Sakamoto KM, Gorr TA, Mehta P, Schmid I, Kropshofer G, Corbacioglu S, Lang PJ, Klein C, Schlegel PG, Heinzmann A, Schneider M, Stary J, van den Heuvel-Eibrink MM, Hasle H, Locatelli F, Sakai D, Archambeault S, Chen L, Russell RC, Sybingco SS, Ohh M, Braun BS (2010). Germline CBL mutations cause developmental abnormalities and predispose to juvenile myelomonocytic leukemia. Nat Genet.

[9] Martinelli S, De Luca A, Stellacci E, Rossi C, Checquolo S, Lepri F, Caputo V, Silvano M, Buscherini F, Consoli F, Ferrara G, Digilio MC, Cavaliere ML, van Hagen JM, Zampino G, van der Burgt I, Ferrero GB, Mazzanti L, Screpanti I, Yntema HG, Nillesen WM, Savarirayan R, Zenker M, Dallapiccola B, Gelb BD, Tartaglia M (2010). Heterozygous germline mutations in the CBL tumor-suppressor gene cause a Noonan syndrome-like phenotype. Am J Hum Genet.

[10] Fang P, Lev-Lehman E, Tsai TF, Matsuura T, Benton CS, Sutcliffe JS, Christian SL, Kubota T, Halley DJ, Meijers-Heijboer H, Langlois S, Graham JM, Beuten J, Willems PJ, Ledbetter DH, Beaudet AL (1999). The spectrum of mutations in UBE3A causing Angelman syndrome. Hum Mol Genet.

[11] Matsuura T, Sutcliffe JS, Fang P, Galjaard RJ, Jiang YH, Benton CS, Rommens JM, Beaudet AL (1997). De novo truncating mutations in E6-AP ubiquitin-protein ligase gene (UBE3A) in Angelman syndrome. Nat Genet.

[12] Cooper TA, Wiggans GR, Null DJ, Hutchison JL, Cole JB (2014). Genomic evaluation, breed identification, and discovery of a haplotype affecting fertility for Ayrshire dairy cattle. J Dairy Sci.

[13] Jansen S, Aigner B, Pausch H, Wysocki M, Eck S, Benet-Pages A, Graf E, Wieland T, Strom TM, Meitinger T, Fries R (2013). Assessment of the genomic variation in a cattle population by re-sequencing of key animals at low to medium coverage. BMC Genomics.

[14] Florea L, Souvorov A, Kalbfleisch TS, Salzberg SL (2011). Genome assembly has a major impact on gene content: a comparison of annotation in two Bos taurus assemblies. PLoS One.

[15] Daetwyler HD, Capitan A, Pausch H, Stothard P, van Binsbergen R, Brondum RF, Liao X, Djari A, Rodriguez SC, Grohs C, Esquerre D, Bouchez O, Rossignol MN, Klopp C, Rocha D, Fritz S, Eggen A, Bowman PJ, Coote D, Chamberlain AJ, Anderson C, VanTassell CP, Hulsegge I, Goddard ME, Guldbrandtsen B, Lund MS, Veerkamp RF, Boichard DA, Fries R, Hayes BJ (2014). Whole-genome sequencing of 234 bulls facilitates mapping of monogenic and complex traits in cattle. Nat Genet.

[16] Zimin AV, Delcher AL, Florea L, Kelley DR, Schatz MC, Puiu D, Hanrahan F, Pertea G, Van Tassell CP, Sonstegard TS, Marcais G, Roberts M, Subramanian P, Yorke JA, Salzberg SL (2009). A whole-genome assembly of the domestic cow, Bos taurus. Genome Biol.

[17] Jung S, Pausch H, Langenmayer MC, Schwarzenbacher H, Majzoub-Altweck M, Gollnick NS, Fries R (2014). A nonsense mutation in PLD4 is associated with a zinc deficiency-like syndrome in Fleckvieh cattle. BMC Genomics.

[18] Gong TW, Huang L, Warner SJ, Lomax MI (2003). Characterization of the human UBE3B gene: structure, expression, evolution, and alternative splicing. Genomics.

[19] Lomax MI, Huang L, Cho Y, Gong TL, Altschuler RA (2000). Differential display and gene arrays to examine auditory plasticity. Hear Res.

[20] Mileski A (2006). Expression Pattern of Ubiquitin Ligase E3 (UBE3B) in Mouse. 35th Annual Meeting and Exhibition of the American Association for Dental Research, 30th Annual Meeting of the Canadian Association for Dental Research and 83rd Annual Meeting and Exhibition of American Dental Education Association.

[21] Yanase S, Ishi N (1999). Cloning of the oxidative stress-responsive genes in Caenorhabditis elegans. J Radiat Res.

[22] Asselbergs FW, Guo Y, van Iperen EP, Sivapalaratnam S, Tragante V, Lanktree MB, Lange LA, Almoguera B, Appelman YE, Barnard J, Baumert J, Beitelshees AL, Bhangale TR, Chen YD, Gaunt TR, Gong Y, Hopewell JC, Johnson T, Kleber ME, Langaee TY, Li M, Li YR, Liu K, McDonough CW, Meijs MF, Middelberg RP, Musunuru K, Nelson CP, O'Connell JR, Padmanabhan S (2012). Large-scale gene-centric meta-analysis across 32 studies identifies multiple lipid loci. Am J Hum Genet.

[23] Porter FD, Herman GE (2011). Malformation syndromes caused by disorders of cholesterol synthesis. J Lipid Res.

[24] Purcell S, Neale B, Todd-Brown K, Thomas L, Ferreira MA, Bender D, Maller J, Sklar P, de Bakker PI, Daly MJ, Sham PC (2007). PLINK: a tool set for whole-genome association and population-based linkage analyses. Am J Hum Genet.

[25] Browning BL, Browning SR (2009). A unified approach to genotype imputation and haplotype-phase inference for large data sets of trios and unrelated individuals. Am J Hum Genet.

[26] Li H, Durbin R (2010). Fast and accurate long-read alignment with Burrows-Wheeler transform. Bioinformatics.

[27] Li H, Handsaker B, Wysoker A, Fennell T, Ruan J, Homer N, Marth G, Abecasis G, Durbin R, 1000 Genome Project Data Processing Subgroup (2009). The sequence alignment/map format and SAMtools. Bioinformatics.

[28] **Picard** http://broadinstitute.github.io/picard/

[29] Quackenbush J, Cho J, Lee D, Liang F, Holt I, Karamycheva S, Parvizi B, Pertea G, Sultana R, White J (2001). The TIGR Gene Indices: analysis of gene transcript sequences in highly sampled eukaryotic species. Nucleic Acids Res.

[30] Gremme G (2005). Engineering a software tool for gene structure prediction in higher organisms. Infor Softw Technol.

[31] Lewis SE, Searle SM, Harris N, Gibson M, Lyer V, Richter J, Wiel C, Bayraktaroglir L, Birney E, Crosby MA, Kaminker JS, Matthews BB, Prochnik SE, Smithy CD, Tupy JL, Rubin GM, Misra S, Mungall CJ, Clamp ME (2002). Apollo: a sequence annotation editor. Genome Biol.

[32] Rozen S, Skaletsky H (2000). Primer3 on the WWW for general users and for biologist programmers. Methods Mol Biol.

[33] **Canadian Dairy Network** http://www.cdn.ca/home.php

[34] Smith PJ, Zhang C, Wang J, Chew SL, Zhang MQ, Krainer AR (2006). An increased specificity score matrix for the prediction of SF2/ASF-specific exonic splicing enhancers. Hum Mol Genet.

[35] Cartegni L, Wang J, Zhu Z, Zhang MQ, Krainer AR (2003). ESEfinder: a web resource to identify exonic splicing enhancers. Nucleic Acids Res.

[36] **ClustalW2** http://www.ebi.ac.uk/Tools/msa/clustalw2/

[37] Kelley LA, Sternberg MJ (2009). Protein structure prediction on the Web: a case study using the Phyre server. Nat Protoc.

