# Supplementary material for: In frame exon skipping in UBE3B is associated with developmental disorders and increased mortality in cattle
Source: BMC Genomics. 2014 Oct 12;15(1):890. doi: 10.1186/1471-2164-15-890 (PMC4203880; doi:10.1186/1471-2164-15-890)
Supplement: Supplementary file 1 — Additional file 1: Table S1: Major clinical features of 18 affected animals. (DOCX 21 KB) [file 12864_2014_6585_MOESM1_ESM.docx]

**Supporting Table 1**

**Major clinical features of 18 affected animals**

| Animal ID | Abnormalities in eyes or eye lids | Intellectual disability apparent | Death within one week of age | Retarded growth after two weeks of age | Aberrant craniofacial appearance | Other abnormalities |
| --- | --- | --- | --- | --- | --- | --- |
| 1 | Present | - | - | Yes | - | Feeding problems |
| 2 | Present | - | - | Yes | - | Feeding problems |
| 3 | Present | Present | Yes | - | - | - |
| 4 | Present | Present | - | Yes | - | - |
| 5 | Present | - | - | Yes | - | - |
| 6 | Present | Present | - | Yes | - | - |
| 7 | Present | - | - | Yes | - | - |
| 8 | Present | Present | - | Yes | Present | - |
| 9 | Present | Present | Yes | - | Present | - |
| 10 | Present | - | - | Yes | - | - |
| 11 | Present | - | - | Yes | - | - |
| 12 | Present | Present | - | Yes | Present | - |
| 13 | Present | - | - | Yes | - | - |
| 14 | Present | Present | - | Yes | - | - |
| 15 | Present | Present | - | Yes | Present | - |
| 16 | Present | Present | - | Yes | Present | - |
| 17 | Present | - | - | Yes | - | - |
| 18 | Present | Present | Yes | - | - | Muscular hypotonia, feeding problems |

- not reported
