# Supplementary material for: In frame exon skipping in UBE3B is associated with developmental disorders and increased mortality in cattle
Source: BMC Genomics. 2014 Oct 12;15(1):890. doi: 10.1186/1471-2164-15-890 (PMC4203880; doi:10.1186/1471-2164-15-890)
Supplement: Supplementary file 2 — Additional file 2: Table S2: Gene content within the segment of extended homozygosity on bovine chromosome 17. The gene content was assessed based on the UMD3.1-assembly of the bovine genome sequence. A total of 14 transcripts/genes were identified within the segment of extended homozygosity. (DOCX 21 KB) [file 12864_2014_6585_MOESM2_ESM.docx]

**Supporting Table 2**

**Gene content within the segment of extended homozygosity on bovine chromosome 17**

| Gene | | Start of translation | Stop of translation | Strand |
| --- | --- | --- | --- | --- |
| Symbol | Full name |  |  |  |
| *GLTP* | glycolipid transfer protein | 65,628,886 | 65,650,280 | + |
| *TRPV4* | transient receptor potential cation channel, subfamily V, member 4 | 65,661,338 | 65,699,848 |  |
| *MVK* | mevalonate kinase | 65,882,632 | 65,863,712 | - |
| *MMAB* | methylmalonic aciduria (cobalamin deficiency) cblB type | 65,882,886 | 65,892,450 | + |
| *UBE3B* | ubiquitin protein ligase E3B | 65,944,427 | 65,903,349 | - |
| *KCTD10* | potassium channel tetramerization domain containing 10 | 65,950,166 | 65,979,499 | + |
| *MYO1H* | myosin IH | 66,022,389 | 65,981,418 | - |
| *FOXN4* | forkhead box N4 | 66,074,400 | 66,097,412 | + |
| *ACACB* | acetyl-CoA carboxylase beta | 66,216,643 | 66,102,468 | - |
| *UNG* | uracil-DNA glycosylase | 66,231,818 | 66,221,123 | - |
| *ALKBH2* | alkB, alkylation repair homolog 2 (E. coli) | 66,234,084 | 66,239,859 | + |
| *USP30* | ubiquitin specific peptidase 30 | 66,263,958 | 66,239,945 | - |
| *SVOP* | SV2 related protein | 66,276,572 | 66,349,821 | + |
| *DAO* | D-amino-acid oxidase | 66,369,059 | 66,356,385 | - |

The gene content was assessed based on the UMD3.1-assembly of the bovine genome sequence. A total of 14 transcripts/genes was identified within the segment of extended homozygosity.
