# Supplementary figures and images for: In frame exon skipping in UBE3B is associated with developmental disorders and increased mortality in cattle
Source: BMC Genomics. 2014 Oct 12;15(1):890. doi: 10.1186/1471-2164-15-890 (PMC4203880; doi:10.1186/1471-2164-15-890)

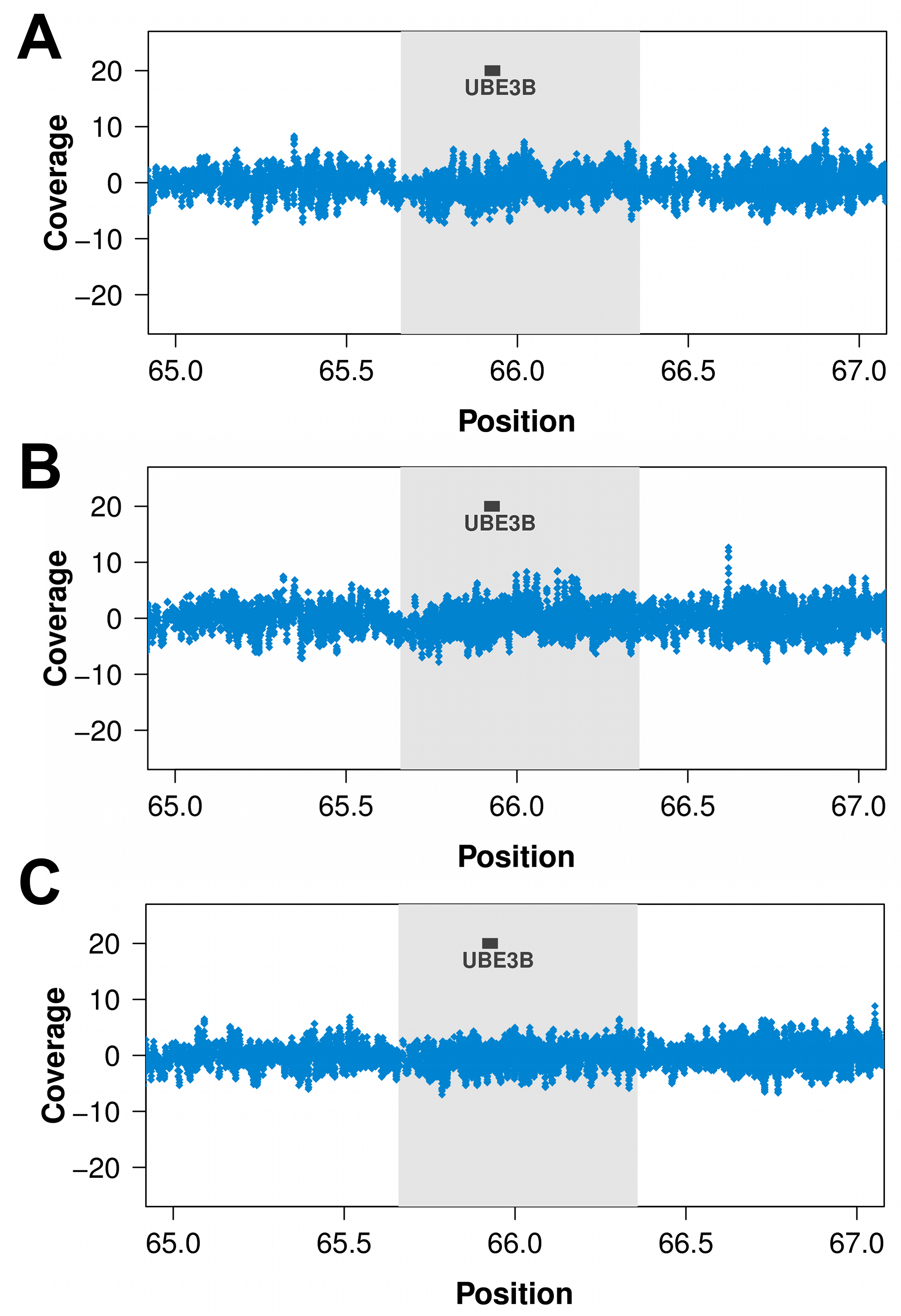

Supplement: Supplementary file 3 — Additional file 3: Figure S1: Average read depth in the PIRM-associated region. Each dot represents the average read depth of 5 adjacent variants displayed as deviation from the average sequence coverage for the affected animal (A), the supposed carrier of the mutation (B) and an unaffected Fleckvieh animal (C). The grey shaded area represents the segment of extended homozygosity including UBE3B. (TIFF 2 MB) [file 12864_2014_6585_MOESM3_ESM.tiff]

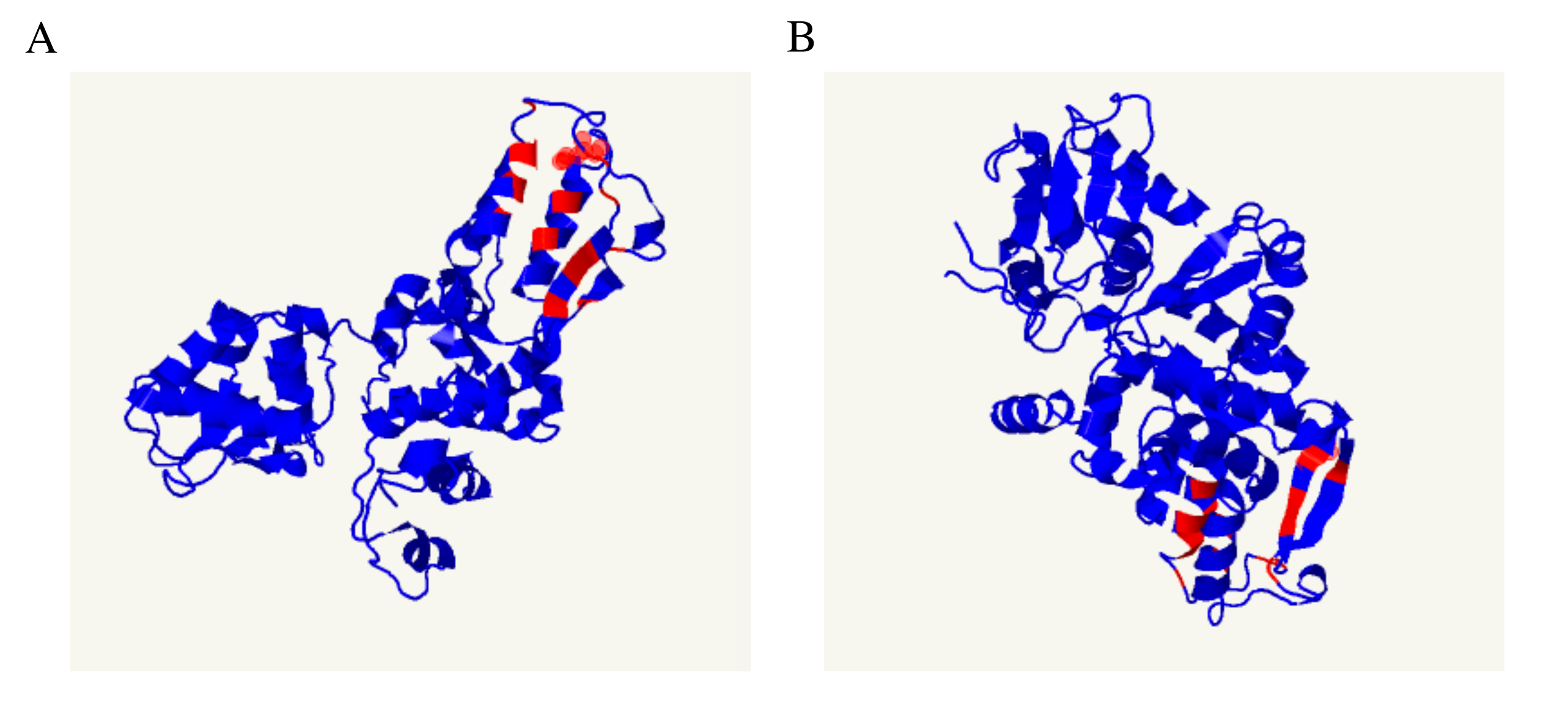

Supplement: Supplementary file 7 — Additional file 7: Figure S2: Three dimensional modelling of the UBE3B HECT domain. The photos show a PHYRE2 analysis of the normal UBE3B protein (A) and lacking 40 amino-acid of which 20 belong to E2 subdomain of HECT (B). E2 subdomain is shown in red. (TIFF 907 KB) [file 12864_2014_6585_MOESM7_ESM.tiff]
