# Supplementary material for: In frame exon skipping in UBE3B is associated with developmental disorders and increased mortality in cattle
Source: BMC Genomics. 2014 Oct 12;15(1):890. doi: 10.1186/1471-2164-15-890 (PMC4203880; doi:10.1186/1471-2164-15-890)
Supplement: Supplementary file 5 — Additional file 5: Table S3: Prediction of the genomic structure of the bovine UBE3B gene. (DOCX 20 KB) [file 12864_2014_6585_MOESM5_ESM.docx]

**Supporting Table 3**

**Prediction of the genomic structure of the bovine UBE3B gene**

| Exon count | Exon start | Exon end |
| --- | --- | --- |
| 1 | 65.949.805 | 65.949.675 |
| 2 | 65.949.674 | 65.949.348 |
| 3 | 65.948.886 | 65.948.882 |
| 4 | 65.947.690 | 65.947.685 |
| 5 | 65.947.195 | 65.947.189 |
| 6 | 65.945.841 | 65.945.739 |
| 7 | 65.944.448 | 65.944.267 |
| 8 | 65.943.752 | 65.943.632 |
| 9 | 65.941.877 | 65.941.818 |
| 10 | 65.941.037 | 65.940.933 |
| 11 | 65.939.413 | 65.939.317 |
| 12 | 65.937.691 | 65.937.606 |
| 13 | 65.936.735 | 65.936.653 |
| 14 | 65.935.494 | 65.935.389 |
| 15 | 65.935.114 | 65.934.994 |
| 16 | 65.934.011 | 65.933.834 |
| 17 | 65.933.395 | 65.933.232 |
| 18 | 65.932.097 | 65.931.930 |
| 19 | 65.927.129 | 65.926.958 |
| 20 | 65.925.407 | 65.925.289 |
| 21 | 65.924.499 | 65.924.385 |
| 22 | 65.923.601 | 65.923.502 |
| 23 | **65.921.616** | **65.921.497** |
| 24 | 65.917.491 | 65.917.315 |
| 25 | 65.917.206 | 65.917.096 |
| 26 | 65.915.285 | 65.915.148 |
| 27 | 65.914.793 | 65.914.728 |
| 28 | 65.911.576 | 65.911.518 |
| 29 | 65.908.708 | 65.908.526 |
| 30 | 65.908.039 | 65.907.928 |
| 31 | 65.906.347 | 65.906.255 |
| 32 | 65.905.418 | 65.904.067 |
