# Supplementary material for: In frame exon skipping in UBE3B is associated with developmental disorders and increased mortality in cattle
Source: BMC Genomics. 2014 Oct 12;15(1):890. doi: 10.1186/1471-2164-15-890 (PMC4203880; doi:10.1186/1471-2164-15-890)
Supplement: Supplementary file 6 — Additional file 6: Table S4: PCR primers. (DOCX 19 KB) [file 12864_2014_6585_MOESM6_ESM.docx]

**Supporting Table 4**

**PCR primers.**

| Primer pair | Sequence | Attach site | Target |
| --- | --- | --- | --- |
| 1. | TGGTGACCAAGGAGAAGGAG | Exon 23 | gDNA |
|  | TGTCCTCGCTGTAGAGGTGA | Intron 23 |  |
| 2. | GCTGCGAAAGGATCTCAAAC | Junction of exons 21 and 22 | cDNA |
|  | ACCACGATTCCCTCGTACAC | Junction of exons 25 and 26 |  |
| 3. | CTCACATCCATCAAGCGGTA | Junction of exons 26 and 27 | cDNA |
|  | CACCGTGTGCTTCTTCAGAT | Junction of exons 29 and 30 |  |
